# Lobo-isthmectomy in the management of differentiated thyroid cancer

**DOI:** 10.1186/s13044-022-00145-1

**Published:** 2023-02-13

**Authors:** Jolanta Krajewska, Aleksandra Kukulska, Konrad Samborski, Agnieszka Czarniecka, Barbara Jarzab

**Affiliations:** 1 Nuclear Medicine and Endocrine Oncology Department, M. Sklodowska-Curie National Research Institute of Oncology Gliwice Branch, Wybrzeze AK 15, 44-102 Gliwice, Poland; 2 Radiotherapy Department, M.Sklodowska-Curie National Research Institute of Oncology Gliwice Branch, Gliwice, Poland; 3 Oncologic and Reconstructive Surgery Clinic, M. Sklodowska-Curie National Research Institute of Oncology Gliwice Branch, Gliwice, Poland

**Keywords:** Differentiated thyroid cancer, Papillary thyroid cancer, Low-risk thyroid cancer, Total thyroidectomy, Thyroid lobectomy, Treatment de-escalation

## Abstract

We have recently witnessed a rapid increase in the incidence of differentiated thyroid carcinoma (DTC), particularly low and very low-risk papillary thyroid carcinoma. Simultaneously, the number of cancer-related deaths has remained stable for more than 30 years. Such an indolent nature and long-term survival prompted researchers and experts to an ongoing discussion on the adequacy of DTC management to avoid, on the one hand, the overtreatment of low-risk cases and, on the other hand, the undertreatment of highly aggressive ones.

The most recent guidelines of the American Thyroid Association (ATA GL) moved primary thyroid surgery in DTC towards a less aggressive approach by making lobectomy an option for patients with intrathyroidal low-risk DTC tumors up to 4 cm in diameter without evidence of extrathyroidal extension or lymph node metastases. It was one of the key changes in DTC management proposed by the ATA in 2015.

Following the introduction of the 2015 ATA GL, the role of thyroid lobectomy in DTC management has slowly become increasingly important. The data coming from analyses of the large databases and retrospective studies prove that a less extensive surgical approach, even if in some reports it was related to a slight increase of the risk of recurrence, did not show a negative impact on disease-specific and overall survival in T1T2N0M0 low-risk DTC. There is no doubt that making thyroid lobectomy an option for low-risk papillary and follicular carcinomas was an essential step toward the de-escalation of treatment in thyroid carcinoma.

This review summarizes the current recommendations and evidence-based data supporting the necessity of de-escalation of primary thyroid surgery in low-risk DTC. It also discusses the controversies raised by introducing new ATA guidelines and tries to resolve some open questions.

## Background

We have recently witnessed a rapid increase in the incidence of differentiated thyroid carcinoma (DTC), particularly low and very low-risk papillary thyroid carcinoma (PTC), showing, in general, the most favorable prognosis among all malignancies [1, 2]. Simultaneously, cancer-related deaths have remained stable for more than 30 years [3]. Such an indolent nature and long-term survival prompted researchers and experts to an ongoing discussion on the adequacy of DTC management to avoid, on the one hand, the overtreatment of low-risk cases and, on the other hand, the undertreatment of highly aggressive ones. This idea has been clearly reflected in the current recommendations of the American Thyroid Association (ATA) [4]. According to Prof. Tuttle’s group, the key changes proposed by the 2015 ATA Guidelines (ATA GL) concerned the management of thyroid nodules, the primary surgery in DTC, and the surveillance following primary DTC treatment [5].

**Fig. 1:**
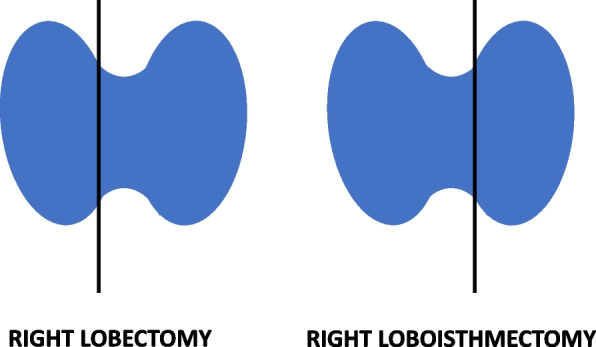
The difference between lobectomy and loboisthmectomy

The most recent ATA GL moved primary thyroid surgery in DTC towards a less aggressive approach by making lobectomy an option for patients with intrathyroidal DTC up to 4 cm in diameter without evidence of extrathyroidal extension or lymph node metastases. On the contrary, in 2009, ATA recommended total or near-total thyroidectomy in nearly all patients preoperatively diagnosed with DTC. The only exception was an intrathyroidal unifocal tumor < 1 cm with no extrathyroidal extension, clinically N0. In such a case, lobectomy was a sufficient procedure [6]. One should note that the surgeons distinguish between thyroid lobectomy and loboisthmectomy. Lobectomy is the removal of one thyroid lobe, whereas the term “loboisthmectomy” concerns the surgical excision of a thyroid lobe together with the isthmus (Fig. 1). In this article, these two terms are treated as equivalent.

The rationale justifying such significant changes in primary thyroid surgery in DTC resulted from careful and critical evaluation of published data comparing clinical outcomes of thyroid lobectomy (TL) and total thyroidectomy (TT), decreasing indications for postoperative RAI treatment and RAI scanning, and finally, the belief that in most cases with persistent or recurrent DTC salvage therapy was quite effective [7]. Although ATA recommendations on surgical management are strong and based on moderate-quality evidence [4], they raised many controversies among surgeons, also in Europe [8].

This paper aims not only to present the role of TL in low-risk DTC management in light of current guidelines but also to summarize the evidence-based data and doubts regarding the limited extent of surgery in DTC. The place of lobectomy in the treatment of papillary thyroid microcarcinoma (PTMC), and ATA changes regarding central lymph node dissection, is not a subject of this review.

### Primary thyroid surgery in DTC in the light of the current guidelines

The current ATA GL (Recommendation # 35) recommends total or near-total thyroidectomy in cases with a primary tumor larger than 4 cm, or with gross extrathyroidal extension, or if clinically apparent lymph node or distant metastases are present. For patients with primary tumor diameter between 1 and 4 cm without extrathyroidal extension, clinically N0 and M0, both bilateral (total or near-total thyroidectomy) and unilateral (lobectomy) procedures are the initial treatment options. Although the guidelines emphasize that “lobectomy may be sufficient for low-risk papillary and follicular carcinomas, the treatment team may choose total thyroidectomy to enable postoperative radioiodine (RAI) therapy, to enhance follow-up based on upon disease features, and/or patient preference” [4].

According to more recent clinical practice guidelines published by the European Society for Medical Oncology (ESMO), TT is still the standard primary surgical approach for DTC other than unifocal PTMC. However, in unifocal T1b-T2 DTC, with no family history of thyroid cancer, no prior head and neck irradiation, no extrathyroidal extension, with the primary tumor not adjacent to the trachea, esophagus, or recurrent laryngeal nerve TL alone is an alternative option [9]. Similar recommendations are proposed by the National Comprehensive Cancer Network (NCCN) [10].

### The rationale supporting lobectomy in low-risk DTC

Surgery plays the most crucial role in DTC management. On the one hand, the completeness of surgery has an undoubtful impact on patient survival and long-term outcomes. On the other hand, any potential postoperative complications, particularly the permanent ones, related to the extent of the surgical procedure may negatively impact the patient’s quality of life.

Previous ATA guidelines [6], published in 2009, endorsed TT in DTC > 1 cm based on data proving its favorable influence on survival and the risk of relapse [11–16]. The broader use of postoperative RAI ablation at that time also impacted the 2009 ATA position. However, more recent [17–22] data led to crucial changes in ATA recommendations.

In 2007 Bilimoria et al. published the results of an analysis of 52,173 PTC patients from the National Cancer Data Base diagnosed between 1995 and 1998. Forty-three thousand, two hundred seventy-seven underwent TT, whereas 8946 patients had a unilateral procedure. The ten-year recurrence rate for tumors > 1 cm was slightly higher in a group subjected to TL compared with a group who underwent TT, 9.8% vs. 7.7%, respectively (p < 0.05). Similarly, 10-year relative overall survival (OS) was slightly lower in patients after TL (97.1%) than in patients after total thyroid resection (98.4%). Also, this difference was statistically significant. The patients were further stratified by tumor size. Considering primary tumors between 1 and 2 cm, patients who had TL demonstrated a 24% higher risk of relapse and 49% higher risk of death than those subjected to TT [23]. However, the authors of this paper did not analyze any other factors than tumor size showing a potential impact on the risk of relapse and cancer-related death. We are not able to find how many patients in this group were diagnosed with low, intermediate, or high-risk PTC. So, we can not exclude that concurrent high-risk features influenced a worse prognosis in the TL group.. These doubts were also raised by Shah in the excellent editorial published in 2008 [24]. The differences reported by Bilimoria et al. disappeared when multivariable adjustment for additional variables related to PTC advancement was made. An updated analysis of 61,775 PTC patients (with primary tumor diameter 1–4 cm) [21] operated on between 1998 and 2006 failed to demonstrate any advantages in survival associated with the extent of surgery. OS did not differ significantly between TT and TL groups (HR 0.96 [0.84–1.09]; *p* = 0.54). Similar results were obtained when the group was stratified by the tumor size: 1–2 cm (HR 1.05 [0.88–1.26]; *p* = 0.61) and 2–4 cm (HR 0.89 [0.73–1.07]; *p* = 0.21). Older age, male gender, black race, tumor size, nodal and distant metastases, and a lower income but not the extent of surgery independently influenced survival in this study [21].

The extent of thyroidectomy was not a major determinant of survival in PTC patients from the Surveillance, Epidemiology, and End Results (SEER) database. This analysis included 4402 patients classified as low-risk based on the Age, Metastases, Extent, and Size (AMES) score. Ten-year survival in patients following TT was comparable to a group treated with partial thyroidectomy, 89% vs. 91%, respectively. Older age, male gender, larger tumor size, N1 feature, and lack of postoperative RAI therapy were among the risk factors associated with higher mortality. Surprisingly, even in 1030 AMES high-risk PTC patients, also evaluated under this study, the differences in survival between partial thyroidectomy and TT were insignificant (78% vs. 72%, respectively) [20]. Two additional studies that analyzed patients from the SEER registry did not demonstrate any advantages of a more extensive surgery either [17, 22]. The first one [17], involved 22,724 PTC patients operated on between 1998 and 2001, among them 5964 patients subjected to TL. In a group of patients with primary tumors ≥ 1 cm, the differences in OS and disease-specific survival (DSS) depending on the extent of surgery were not significant [17]. The second one [22], included 23,605 DTC patients who underwent thyroid surgery between 1983 and 2002 (12,598 patients – TT and 3266 patients – TL). The 10-year survival rates for TT and TL were 90.4% and 90.8%, respectively [22].

Two other single-center studies [18, 19], considered by ATA, were published shortly before the introduction of the guidelines. The Japanese retrospective analysis summarized 1088 PTC cases after TL with curative intent. The cause-specific survival rate after a median follow-up of 17 years was 98%. Based on these data, the authors concluded that TL could be an alternative to TT in patients younger than 45 years, with a primary tumor 4 cm or less without extrathyroidal extension and lymph node involvement [18]. Nixon et al. reported a group of 889 patients with T1-T2 intrathyroidal DTC. Fifty-nine percent of them (528 patients) had TT, and 41% (361 patients) had TL. According to the multivariate analysis, age over 45 years and male gender were independent predictors of poorer survival, but not the type of surgery and tumor size. There wasn’t any difference in the risk of local and regional recurrence depending on the extent of thyroid surgery [19].

More recent studies also support the idea of “less is more” in thyroid surgery in DTC. In 2017 Kuba et al. retrospectively summarized Japanese experiences. This study involved 173 PTC patients, clinically N0 and M0. The size of the primary tumor ranged between 1 and 5 cm. Fifty-three patients underwent TT, whereas the remaining 120 patients had TL. After adjustment for clinicopathological factors, no significant differences in recurrent-free survival (RFS) and OS were reported. Ten-year RFS in patients treated with TL and TT were 93.0% and 90.6%, while OS was 96.9% and 96.2%, respectively [25]. A Korean group reported similar results. This analysis enrolled 2345 PTC cases, with thyroid tumors 1–4 cm, without nodal and distant spread. In the matched pair analysis, disease-free survival (DFS) rates were comparable in TL and TT groups, also when stratification by tumor size (1–2 cm and 2–4 cm) was applied [26]. On the contrary, the other study from Korea, which involved a group of 5266 patients with primary tumor diameter 1–4 cm (mean size 1.84 cm ± 0.74), 97.5% with PTC, and 2.5% with follicular thyroid cancer (FTC), showed that TT reduced the rate of recurrences compared to TL. The extrathyroidal extension was diagnosed in 69.3% of patients, 35.1% had multifocal tumors, 26.4% had bilateral disease, 53% had central lymph node metastases, and 19.9% had lateral lymph node metastases. TT was performed in 4292 patients, and TL in 974 patients. The recurrence rate in the TT group was 5.7%, whereas in the TL group – 9.4%. The differences in DFS between the groups reached statistical significance. Ten-year and 20-year DFS in TT and TL groups were 93.8% and 92.4%, and 89% and 83.6%, respectively. One should notice that this cohort includes risk features that are not currently indications for lobectomy, such as an extrathyroidal extension or lateral lymph node metastases. However, the TL group demonstrated a slightly better DSS than the TT group – 25-year DSS were 100% in TL and 99.6% in TT. This difference was also significant [27]. These data demonstrate that even if a higher recurrence rate is related to the less extensive surgical approach, it does not significantly impact the risk of cancer-related death.

A systematic meta-analysis of 16 studies (some of them described above [21, 23, 25]) with 175,430 DTC patients with a primary tumor up to 4 cm limited to the thyroid was published in 2020. The type of thyroid surgery did not lead to significant differences in OS, DFS, and DSS. The median recurrence rate was the same in TL and TT groups – 7%. Similarly, OS in both groups was 93% [28].

The two most recent papers were published in 2021. The first one was a retrospective cohort study of a group of 295 patients treated with thyroid lobectomy, followed by a mean of 19.1 years. The majority of patients were diagnosed with PTC (89.2%). The remaining ones had FTC. The mean tumor size was 22.9 mm ± 16.9 mm. According to the ATA risk stratification, 91.9% of patients were classified as low-risk, whereas 8.1% as intermediate-risk. Fifty-four patients (18.3%) required reoperation. Recurrence was confirmed in 40 (13.6%) cases. In 55% of them, DTC recurrence was diagnosed more than 10 years after primary surgery. At the final assessment, 282 patients (95.6%) were disease-free. DTC relapse was more frequent in patients with aggressive histology (19.2% vs. 4.1%) and intermediate-risk ones (28.6% vs. 7.1%). Tumors > 4 cm found in 11.5% of cases were associated with a significantly higher risk of relapse and lower remission rate than the smaller ones [29]. The second paper presents a single-institution study using propensity score (PS) matching analysis to compare TL and TT carried out in a group of 3756 DTC patients staged T1T2N0/Nx. They were stratified as low or intermediate risk. The main inclusion criteria involved intrathyroidal tumor < 4 cm, no nodules in the contralateral lobe, no suspicious central lymph nodes in preoperative assessment, and/or intraoperative palpation. TL was performed in 943 patients, whereas the remaining 2813 patients underwent TT. Age, sex, histology, RAI therapy, ATA risk class, and TNM stage were selected as PS matching criteria to reduce potential bias and control possible confounders. After PS matching, no significant differences between TL and TT for OS, DSS, and RFS were found [30]. Both papers confirm that TL may be a therapeutic option for low-risk T1T2 patients.

One may also find some papers not enhancing a less extensive surgical approach in low-risk DTC, particularly in T2 tumors (2–4 cm).

Rajjoub et al*.* evaluated 33,816 PTC patients registered in the National Cancer Data Base, operated on between the years 2004 and 2008. The majority of the patients (22,899) were diagnosed with classic PTC. In the remaining 10,918 cases follicular PTC variant was confirmed. The study enrolled N0 and M0 patients with tumor sizes ranging between 1–3.9 cm. Multifocality, extrathyroidal extension, and radiation history were among the exclusion criteria. TL was performed in 2,835 patients, whereas 30,981 patients underwent TT. Adjusted analysis for patients and clinical factors showed that in cases with classic PTC variant and tumor diameter 2–3.9 cm, TT was associated with significantly improved OS compared to TL. Regarding a group diagnosed with follicular PTC variant, no significant differences in OS between TT and TL were noticed [31].

A systematic review and metaanalysis compared the survival or recurrence concerning surgical management: TL vs. TT. It included 13 studies carried out in PTC, published between 2005 and 2018. Seven out of these 13 studies evaluated the impact of the extent of the surgery on OS. No differences were found between TL and TT groups if the tumor size was 1–2 cm. However, in larger tumors, between 2 and 4 cm, TT was associated with significantly better OS than TL (HR 0.88 [0.79–0.99]; p = 0.03). Metaanalysis for RFS, based on nine studies, demonstrated that patients treated with TT had significantly better RFS than those treated with TL (HR 0.56 [0.41–0.77] *p* < 0.0001). The difference between TT and TL groups was also significant when tumors > 1 cm were analyzed separately [32].

The data presented in this chapter are summarized in Table 1.

**Table 1:** Summary of the data on thyroid lobectomy in low-risk differentiated thyroid carcinoma

Study	Number of patients	Source	Main outcomes
Studies that ATA took into account preparing 2015 ATA GL			
Mendelsohn [17]	22,724	SEER	No difference in survival between TT and TL
Matsuzu [18]	1088	Single center	All treated with TL. No comparison between TT and TL 25-year DSS 95.2%
Nixon [19]	889	Single center	No difference in local and regional recurrence between TT and TL No differences in OS and DSS
Haigh [20]	5432	SEER	No differences in survival between TT and TL
Bilimoria [23]	52,173	NCDB	Significantly higher risk of recurrence and death in tumors > 1 cm after TL than TT
Adam [21]	61,775	NCDB	No difference in OS between TT and TL
Barney [22]	23,605	SEER	No differences in OS and DSS between TT and TL
Studies published after the introduction of 2015 ATA GL			
Kuba [25]	173	Single center	No differences in RFS and OS between TT and TL
Song [26]	2345	Single center	No difference in DFS between TT and TL
Choi [27]	5266	Single center	Significantly lower DFS after TL than TT. No difference in DSS between TT and TL
Bosset [29]	295	Single center	All treated with TL. No comparison between TT and TL. Recurrence rate 13.6%
Matsuura [30]	6259	Single center	No differences in OS, DSS, and RFS between TT and TL
Rajjoub [31]	33,816	NCDB	TT compared with TL associated with significantly improved survival in tumors 2–3.9 cm, no difference in tumors 1–1.9 cm

*ATA GL* The guidelines of the American Thyroid Association, *TT* Total thyroidectomy, *TL* Thyroid lobectomy, *SEER* Surveillance, Epidemiology and End Results, *NCDB* National Cancer Database, *OS* overall survival, *DSS* disease-specific survival, *RFS* regional-free survival, *DFS* disease-free survival

### How did 2015 ATA GL affect daily clinical practice ?

Some data answering this question have been published since the introduction of 2015 ATA GL. A group of 118 DTC patients operated on between 2013 and 2014 and 51 DTC patients who underwent thyroid surgery in 2016 were subjected to a retrospective study. Patients with primary tumors larger than 4 cm, multifocal tumors, or lymph node involvement were excluded from the analysis. The mean primary tumor size did not differ significantly between the groups. It was 1.8 cm and 2.1 cm, respectively. Total thyroidectomy was done in 72 (61%) patients treated before and in 16 (31.4%) patients operated on after the introduction of 2015 ATA GL. What is more important, the rate of completion thyroidectomy, although a bit higher than recommended by ATA, significantly decreased in 2016 compared with the earlier period 2013–2014, 73,9% vs. 20%, respectively [33].

The results of another retrospective analysis come from a tertiary center in Australia. The study group involved 339 patients preoperatively diagnosed with Bethesda V or VI category in the thyroid nodule 1–4 cm in diameter without clinically apparent nodal or distant metastases. One hundred eighty-six patients (54.9%) underwent thyroid surgery between 2010 and 2015, whereas the remaining 153 patients (45.1%) between the years 2016–2019. Following the 2015 ATA GL, the rate of hemithyroidectomy significantly increased from 5.4% to 19.6%. Simultaneously, the ratio of completion thyroidectomy dropped from 50% up to 27.6%. However, the difference between pre-ATA2015 and post-ATA 2015 did not reach statistical significance. Smaller tumor size, younger age, and Bethesda V category were independently associated with an increased likelihood of thyroid lobectomy [34].

Relevant data on surgical treatment in low-risk PTC resulted from the Collaborative Endocrine Surgery Quality Improvement Program (CESQIP), which captures approximately 2.4% of all thyroid operations in the United States. A group of 740 PTC tumors < 4 cm operated between 2014 and 2019 and subjected to retrospective analysis was selected among 18,881 cases from the CESQIP database. Thyroid lobectomy was carried out in 117 (15.8%) patients, whereas the remaining 623 patients underwent total thyroidectomy. The number of lobectomies decreased with the tumor size. Lobectomy was performed in 15.8% of patients with PTC tumors between 1 and 2 cm and only in 6,1% with tumor diameter between 2–4 cm. Interestingly, high variations between sites were observed. High-volume centers showed a slightly higher rate of lobectomies than low-volume sites. Prior to the release of 2015 ATA GL, only 3.7% of patients with PTC tumors < 4 cm had thyroid lobectomy. Following the 2015 ATA GL, the percentage of patients qualified for the unilateral procedure was 21.9%. Although these results demonstrated a statistically significant trend towards more thyroid lobectomies during the study period, total thyroidectomy remained the most frequent primary surgical procedure in the US, even in PTMC – 75.8% of patients [35]. Nevertheless, this study clearly reflects the impact of 2015 ATA GL on clinical practice in the US, particularly when we compare it with a study summarizing the data obtained from the SEER registry between the years 2000 and 2014. During this period, 44,537 PTC patients underwent thyroid surgery. The percentage of total thyroidectomies in 2000 was 78,1%. This ratio increased in 2014 to 85.7%. Simultaneously, the proportion of lobectomy dropped from 16.6% in 2000 to 11.4% in 2014 despite increasing worldwide incidence of low-risk PTC [36].

Contrary to the above-mentioned studies, the data reported by a medium–high volume Italian thyroid surgery center in an endemic region did not demonstrate any impact of 2015 ATA GL on daily clinical practice. Only three out of 197 patients subjected to thyroid surgery underwent TL. The main reason favoring TT were tumor multifocality and tumor size [37]. A similar opinion was presented by another Italian group. The indication for lobectomy might be limited in endemic areas by the coexistence of other thyroid conditions [38].

### Controversies related to “less is more” in primary thyroid surgery in low-risk DTC

The 2015 ATA GL raised many controversies regarding primary thyroid surgical management. According to European experts, 43% of lobectomized patients may require completion thyroidectomy because of risk factors present only in the histopathological examination, lobectomy precludes postoperative RAI therapy, the need for postoperative thyroxine use in Europe is much higher than expected by the guidelines, and finally, the evidence supporting lobectomy is based on large databases, which may have accuracy issues [39], whereas “a key study [21] favoring lobectomy may have been subject to bias or inaccuracy” [8].

In another paper on personalized management of DTC in real practice, a multidisciplinary panel of European experts emphasized that “more evidence is necessary before generalizing the current trend towards the use of lobectomy for low-risk primary tumors ≤ 4 cm”. In their opinion, it may be safer to limit the primary tumor diameter to < 2 cm in low-risk patients referred to TL [40].

a) Postoperative RAI therapy.

Since the indication for routine use of postoperative thyroid RAI therapy, especially in low-risk DTC, was substantially limited [4, 9, 10], the major reason supporting total thyroidectomy in these patients seems to be no longer crucial.

b) Completion thyroidectomy – in whom  it is necessary ?

An important argument raised by opponents of thyroid lobectomy is the potential necessity of completion thyroidectomy (CT) in patients in whom a histopathological examination reveals additional risk factors. According to the authors of 2015 ATA GL, with the proper patient selection, CT rates < 10% following TL may be achieved [4]. The European experts estimated a much higher percentage of CT – 43% [8]. Their assumption was confirmed by the data published by Sawant et al. in 2019. The study evaluated a group of 361 DTC patients diagnosed between 2009 and 2015. Forty-five percent of them (161 patients) required CT [41]. The main reason for CT was the presence of indications for postoperative RAI therapy, recommended by the British Thyroid Association Guidelines in the majority of DTC patients at this time. The authors concluded that in light of current recommendations limiting the postoperative use of RAI therapy, more patients would be eligible for a more conservative approach. Similar data were reported by the Australian group. According to preoperative evaluation, 275 thyroid tumors were classified as low-risk and fulfilled the ATA criteria for TL. However, based on postoperative histopathological evaluation, 42.5% of tumors represented requirements for CT. The most common reasons were angioinvasion alone, local invasion alone, greater than five involved central lymph nodes, and the presence of multiple factors (40.2% of patients referred to CT) [42].

On the other hand, one should notice that the CT rate reported by two centers, which shared their experiences with new ATA GL, was substantially lower than estimated by the European experts, 20% and 27.6% [33, 34]. In contrast, the rate of CT reported by Memorial Sloan Kettering Cancer Center ranged only between 5 and 10% [43].

An important issue that was used to influence the decision-making on qualification for CT was tumor multifocality, which is quite common in PTC. The data on its role as a risk factor for disease recurrence are inconsistent [44–47]. The analysis of 3296 conventional PTC patients with tumor size 1–4 cm, treated with total thyroidectomy, revealed that per 10-year age increment, the presence of *BRAF* mutation, multifocality, and N1b feature were independent factors for bilaterality [48]. Based on the metaanalysis of 8 studies (4347 PTC patients), the prevalence of occult PTC in the contralateral lobe was 26.6%. A tumor size > 1 cm, ipsilateral multifocality, contralateral benign lesions, and lymph node metastases in the central neck compartment showed a significant association with contralateral occult PTC [49]. Some studies were carried out to answer whether multifocality should be an indication for CT. One hundred and fifty PTC patients, among them 41 low-risk cases, entered into a prospective tumor registry between the years 1985 and 2005. They underwent TL before having a definite diagnosis of thyroid cancer. Initial resection of a thyroid lobe was followed by CT and RAI therapy. Forty-one percent of patients had a contralateral disease. No differences in survival and recurrence rate between patients with and without contralateral disease were observed [50]. One should remember that the diagnosis of thyroid cancer itself was a sufficient reason for CT at the time when these patients were diagnosed. Similarly, postoperative RAI ablation used to be also an everyday routine and simultaneously an additional justification for CT. The most important finding resulting from this study is the lack of substantial impact of contralateral disease on long-term PTC prognosis. A more recent analysis by Harries et al. was reported in 2020. It was a retrospective study of 849 DTC patients initially managed with a lobectomy. Six hundred nineteen patients had a unifocal disease and 230 multifocal DTC. PTC in the contralateral lobe was diagnosed during the study period in 5 patients with multifocal tumors and 9 patients with unifocal tumors. The authors calculated 5- and 10-year contralateral lobe PTC-free probability, which was 97.8% and 97.8% in patients with multifocal tumors and 99.1% and 98,6% in cases with the unifocal disease. This difference was insignificant. No significant differences in regional recurrence-free probability between multifocal and unifocal diseases were observed. The ten-year rates were 99.4% and 99.5%, respectively. There were no disease-specific deaths in both groups, whereas 10-year OS was 93.1% in patients with multifocal tumors and 91.6% with unifocal tumors [51]. The results of both papers speak against routine CT in patients with multifocal disease.

Another question is whether minimal extrathyroidal extension (mETE) constitutes an absolute indication for total thyroid resection or CT. According to the 2015 ATA GL, mETE classifies DTC as intermediate-risk, more routinely qualified for complementary RAI therapy. However, the 8th edition of the AJCC / UICC TNM classification [52] does not consider the presence of mETE as a factor affecting primary DTC staging and prognosis. The rationale for this change is illustrated by a meta-analysis, which included 23,816 patients from 13 retrospective studies. When studies that included N0 and N1 patients were analyzed, the risk of recurrence in the group with mETE was 7%, whereas in the group without mETE – 6.2%. This difference was statistically significant. In DTC patients without lymph node metastasis, mETE was associated with a statistically significant increase in the risk of DTC recurrence (3.5%) compared with patients in whom mETE was not observed (2.2%). However, the absolute increase in risk was slight, being < 5% for patients without node involvement, so within the low-risk group's accepted range. There was no association between mETE and the risk of death from DTC [53]. Similarly, a Korean retrospective analysis of 734 PTC patients demonstrated a positive correlation between mETE and RFS [54]. Interestingly, other studies demonstrated no effect of mETE on the risk of DTC recurrence [55, 56], regardless of whether the primary tumor diameter was smaller or larger than 4 cm [57]. The 10-year recurrence-free survival in N0,M0 patients whose primary tumor diameter was ≤ 4 cm was 90%, 82%, and 83% in patients without ETE, with mETE limited to perithyroid soft tissues, and with gross ETE, respectively. However, age > 55 years, male sex, and lymph node metastases rather than ETE were the predictive factors significantly associated with worse DFS in multivariate analysis. OS and DSS did not significantly differ between groups [58].

c) The need for thyroxine supplementation following lobectomy.

No need for postoperative thyroxine supplementation may be one of the advantages of a less extensive surgical approach. Although frequently shared by the supporters of TL, such an opinion has been called into question by European thyroidologists [8]. Numerous data demonstrated these doubts are reasonable [59–62]. TSH level above 2mIU/L was observed in 85% of patients 6 weeks after TL due to low-risk PTC. This percentage increased up to 88% by 3–6 months [61]. Thyroid hormone supplementation after TL is more frequently required in patients diagnosed with thyroid cancer than with benign goiter, 73% and 38%, respectively [60]. The cut-off value of preoperative TSH level of 1.7 mIU/L allowed predicting postoperative TSH increase > 2mIU/L. Multivariate analysis demonstrated that a preoperative TSH level was the only independent risk factor related to a postoperative TSH increase > 2mIU/L [61]. According to the Korean group, not only a high preoperative TSH was a risk factor for postoperative hypothyroidism. Also, a low free serum thyroxine level was significantly associated with the need for postoperative thyroid hormone supplementation [62]. The above-mentioned findings were confirmed in a prospective study, which demonstrated that the risk of hypothyroidism post TL depends on preoperative TSH level and the lower ratio of the weight of thyroid remnant to the patient’s weight [63]. These data prove that most DTC patients will require thyroxine administration after TL even if their TSH level is below the upper limit of 4 mIU/L. One should notice that the upper TSH limit for low-risk DTC patients recommended by the ATA GL is 2mIU/L [4]. According to Zatelli et al., the development of hypothyroidism following TL was significantly associated with a preoperative TSH level higher than 2.43 mIU/L. The proposed cut-off value showed a specificity, sensitivity, positive, and negative predictive value of 93.9%, 31.7%, 91.4%, and 40%, respectively [64]. Thirty-six percent of patients recovered to the euthyroid state within the first year after surgery. A preoperative serum TSH level was significantly associated with the recovery [65]. On the contrary, 66% of patients required thyroxine supplementation. The incidence of hypothyroidism increased until 3 years and remained stable in the 4th and 5th years after surgery [66].

d) Postoperative complications and the extent of surgery.

The extent of surgery is related to the risk of postoperative complications. Also, the reason for surgery demonstrated an impact on the risk of complications. The frequency of recurrent laryngeal nerve injury in patients operated on due to thyroid cancer was higher than in those operated on because of benign goiter. Similarly, the risk of postoperative hypocalcemia was significantly higher after the operation for thyroid cancer than benign conditions [67, 68]. On the contrary, the other report showed that TT due to Graves disease had the highest percentage of complications (17.5%), even higher than due to thyroid carcinoma (13.2%) [69]. It may be explained by the fact that high-volume surgeons more often operated on thyroid cancers than benign conditions [70]. TT was not only related to a significantly higher risk of hypocalcemia and recurrent nerve injury but also to a meaningfully increased risk of bleeding, hematoma, respiratory complications, and tracheostomy than TL [71]. The rate of postoperative surgical complication is also relatively high after complete thyroidectomy, even up to 14%, as reported by Sawant et al. from a high-volume group [41]. The authors conclude that due to such a high frequency of CT-related complications, it should be considered only in those patients “who were most likely to benefit from further surgery to facilitate adjuvant RAI” [41].

Obviously, the risk of postoperative complications also depends on the surgeons’ experience, and the difference between high-volume and low-volume surgeons could be even statistically significant [68, 71]. The high-volume surgeons had a significantly shorter length of hospitalization, a lower incidence of recurrent laryngeal nerve injury and hypocalcemia, and in-hospital deaths than the low-volume ones [67, 68, 70, 71]. Nevertheless, even in the hands of a high-volume surgeon, TT is associated with an increased risk of complications than a less extensive procedure. The total risk of complications following TT and TL for high-volume and low-volume surgeons was 14.5 and 7.6%, and 24.1% and 11.8%, respectively [71].

Although a rare complication, recurrent nerve injury may lead to numerous consequences, including lower respiratory tract infection, emergency readmission to hospital, presence of dysphagia, and tracheostomy or gastrostomy placement [72]. Male sex, age, TT, and postoperative bleeding or iatrogenic complications were risk factors for late vocal palsy. On the contrary, annual surgeon volume > 30 thyroid surgeries was an independent protective factor [72]. An analysis of 11,370 patients from the American College of Surgeons National Surgical Quality Improvement Program thyroidectomy-specific database demonstrated that the frequency of recurrent nerve injury depended on the primary indication for surgery and its extent. It was diagnosed in 4,3% of patients operated on due to a single thyroid nodule and in 9% of patients who underwent surgery due to thyroid cancer. It occurred significantly more often in patients after TT than TL, 6.9% vs. 4.3%, respectively, and in surgeries without intraoperative nerve monitoring – 6.5% vs. 5.6%, respectively [73].

Hypoparathyroidism is the most common complication related to thyroid surgery, more frequently associated with TT than TL. According to US data, based on the analysis of 62,722 procedures, the risk of postoperative hypocalcemia was 16.7% following TT, whereas after TL – 7.1%. This difference was significant [71]. The rate of postoperative hypocalcemia varied depending on the surgeon’s experience, between 4.7% in high-volume surgeons and 12.1% in low-volume ones [69]. In most patients, it recovers within the first year following thyroidectomy. Undetectable serum PTH on postoperative day one was an independent risk factor for permanent hypoparathyroidism [74]. Permanent hypoparathyroidism, defined as a persistent need for vitamin D and/or calcium supplementation at one year postoperatively, is related to increased long-term morbidity and mortality [75, 76]. The frequency of permanent hypoparathyroidism ranges between 1,9%—12.5% [77–81]. Among independent risk factors for permanent postoperative hypoparathyroidism, incidental parathyroidectomy, the extent of thyroid surgery, surgeon experience, age above 60, and female gender were reported [77, 79, 82]. It has been shown recently that patients with permanent hypoparathyroidism following thyroidectomy were characterized by a reduced median mental score ratio and a lower voice quality. Their quality of life, evaluated based on the validated SF-36 questionnaire, was significantly reduced compared to the controls [83]. Global health, physical functioning, role functioning, emotional functioning, and insomnia were domains affected in patients with prolonged calcium or vitamin D intake when compared to those without intake [84]. According to the analysis of 4828 patients from the Scandinavian Quality Register for Thyroid, Parathyroid, and Adrenal surgery, permanent hypoparathyroidism after thyroid surgery for benign conditions significantly increased the risk of renal insufficiency (hazard ratio 4.88 [2.00–11.95]) and any malignancy (hazard ratio 2.15 [1.08–3.47]). Patients with permanent hypoparathyroidism and with known cardiovascular disease prior to surgery had an increased risk of cardiovascular events [75]. What is even more important, it was demonstrated that patients with permanent hypothyroidism after total thyroidectomy due to benign disease had an increased risk of death (hazard ratio 2.09 [1.04–4.2]) [76].

e) Serum thyroglobulin in the follow-up of DTC patients after lobectomy.

Difficulties in the use of serum thyroglobulin (Tg) evaluation in DTC follow-up after TL is an issue raised by the supporters of total thyroidectomy, as lobectomy may create some challenges in using and interpreting Tg and Tg antibodies (antiTg). The 2015 ATA GL proposed new criteria for classifying treatment outcomes based on, among others, serum Tg level and antiTg. These criteria referred only to the patients who underwent total thyroidectomy and postoperative RAI therapy [4]. Shortly thereafter, a paper by Momesso et al. worked up the requirements for excellent, incomplete biochemical, incomplete structural, and indeterminate response showing how to interpret serum Tg and Tg antibodies level as well as imaging studies in patients after TT alone and TL [85]. The absolute values of both stimulated and nonstimulated Tg levels were higher than expected for patients who underwent total thyroid resection and RAI ablation. Tg dynamics was rather more important. One may note that the current ESMO Guidelines endorse some of the cutoffs proposed by Momesso and others. However, some published data demonstrated a limited value of serum Tg and antiTg monitoring in predicting or detecting DTC recurrence in patients after TL [86, 87]. Neck ultrasound also plays an important role in follow-up. It is even more crucial than Tg assessment.

f) Surgical volume and the extent of the surgery.

The questions of what influences surgeons' choice and whether their experience in thyroidectomy plays a role in treatment decision-making seem to be justified. The published data confirmed that high-volume surgeons were more likely to perform total thyroidectomy [67, 70, 71, 88, 89]. Nevertheless, in the US, thyroidectomies are more often performed by low-volume surgeons than by high-volume ones [71].

The French study based on the analysis of a Nationwide Cohort of 375,810 patients demonstrated that, among others, the number of procedures per year was an independent factor influencing the extent of thyroidectomy. A total thyroidectomy was more frequently performed in centers with a caseload > 40/year [88]. What is more important, the number of thyroid procedures carried out by high-volume surgeons significantly increased through the years from 12%-15.7% in the nineties up to 25%-30.9% in the first decade of the twenty-first century [67, 70]. Following the publication of the 2015 ATA GL, the number of thyroid lobectomies showed an increased trend in high-volume centers [35, 90]. Valuable data resulted from a National Survey carried out in the United States among the 320 surgeons registered with the American Medical Association. The majority (64.4%) of participants were low-volume surgeons (< 26 thyroidectomies/year). Surprisingly, it was the high-volume surgeons who more frequently recommended lobectomy for low-risk PTC measuring 1.1–3 cm. However, the proportion of surgeons opting for TL in low-risk PTC 1.1 to 4 cm in size ranged from 43.1% – 2.6%. According to one-third of the responders, TL is underused, while 10% of them believe that it is overused. Regarding low-volume surgeons, they were less likely to use clinical practice guidelines or to be aware guidelines support TL [90]. Similar data were published by Wrenn et al. The proportion of TLs has gradually increased since the introduction of the 2015 ATA GL, mainly in high-volume centers. However, TT remained the most common surgical procedure for thyroid carcinoma [35].

Finally, surgeon experience and the associated risk of complications due to the length of hospital stay and the treatment required have real economic implications for the health system [89, 91].

f) How to preoperatively diagnose low-risk DTC ?

The most crucial issue in the decision-making process on the extent of surgery is a cautious preoperative evaluation of all risk factors. This assessment is based mainly on neck ultrasound and cytopathological evaluation. However, a recent analysis published by an Italian group demonstrated that preoperative thyroid ultrasound failed to reliably exclude tumor multifocality and ETE. The positive predictive value 57.14% and 41.67% regarding multifocality and ETE, respectively, whereas negative predictive values were 63.2% and 72.7% [92]. Thus, in some cases, additional imaging studies may be required. A potential role of molecular markers may be considered. Unfortunately, no reliable molecular markers have been established to support preoperative risk stratification. According to Dhir et al., it was difficult to predict, based on the 2015 ATA GL, the correct extent of thyroidectomy in patients diagnosed with thyroid cancer by a fine needle aspiration biopsy. Among 307 patients suitable for TL by 2015 ATA GL, the postoperative histopathology identified intermediate-risk DTC in 175 (59%) patients. Based on the preoperative assessment, intermediate-risk PTC, in comparison with low-risk PTC, was associated with age ≥ 45 years, female gender, larger tumor size, and the presence of BRAF mutation or RET/PTC rearrangements [93]. Fifty-nine percent of patients from this study had an extrathyroidal extension. Due to recent data proving the lack of its negative impact on survival, it is not involved as a prognostic factor in the current TNM classification. It seems likely that it will cease to be an indication for CT and RAI ablation in the future. Other data demonstrated that intermediate or high-risk features could be postoperatively detected even in up to 42.8% of patients eligible for TL as the initial procedure. They mainly involved the presence of aggressive histology, vascular invasion, extrathyroidal extension, positive margins, or positive lymph nodes [94–96]. Some of the risk factors may be detected by surgeons intraoperatively even in 21% of patients qualified for TL and change the extent of thyroid surgery for TT during the initial procedure [97].

g) Patient perspective.

Finally, a very important issue is how the extent of surgery looks from the patient perspective. Interesting data have been provided by an analysis of the PROFILES Registry data. Based on the EORTC-QlQ-C30 questionnaire, there were no significant differences in global health, physical functioning, role functioning, emotional functioning, cognitive functioning, and social functioning between patients who underwent TL and TT. Compared with the TL group, patients treated with total thyroidectomy demonstrated significantly firmer belief in the necessity of their medication and greater concerns about taking their medication. Patients from the TT group significantly more frequently “agree” or “strongly agree”, among others, with the following statements: “my life will be impossible without medications”, “my health, at present, depends on my medicines”, or “without medicine, I become very ill” [98]. According to the other data, patients who underwent TT were 1.5 times more likely to experience health-related quality of life issues than patients treated with TL [99].

Participants of online surgery were ready to accept a 4.1% risk of relapse if the risk of second surgery would be reduced from 40 to 10%. They also would take a 1.6% risk of cancer recurrence if the risk of postoperative calcium disturbances could be reduced from 3 to 0% [100]. Thirty-nine percent of 1,546 patients participating in another online survey admitted that they would have chosen TL instead of TT if there had been no change in overall DTC outcome. More importantly, 64% of responders preferred more time with their clinicians when the extent of surgery was considered [101]. This paper confirms that so-called shared decision-making, already used in other malignancies, is also a way for patients diagnosed with thyroid carcinoma, especially since the physicians' preference is strongly associated with their recommendation and influences the patient's decision [102].

### Summary and conclusions

Following the introduction of the 2015 ATA GL, the role of thyroid lobectomy in DTC management has slowly become increasingly important. The data coming from analyses of the large databases and retrospective studies prove that a less extensive surgical approach, even if in some reports it was related to a slight increase in the risk of recurrence, did not show a negative impact on disease-specific and overall survival in T1T2N0M0 low-risk DTC. There is no doubt that making thyroid lobectomy an option for low-risk papillary and follicular carcinomas was an important step toward the de-escalation of treatment in thyroid carcinoma.

## Data Availability

Not applicable.

## Abbreviations

AJCC: American Joint Committee on Cancer
AMES: Age, metastases, extent, and size risk classification
AntiTg: Antithyroglobulin antibodies
ATA: American Thyroid Association
ATA GL: 2015 ATA Guidelines on Thyroid Nodules and Differentiated Thyroid Cancer
CT: Completion thyroidectomy
CESQIP: Collaborative Endocrine Surgery Quality Improvement Program
DFS: Disease-free survival
DSS: Disease-specific survival
DTC: Differentiated thyroid carcinoma
ESMO: European Society for Medical Oncology
mETE: Minimal extrathyroid extension
NCCN: National Comprehensive Cancer Network
OS: Overall survival
PS: Propensity score
PTC: Papillary thyroid carcinoma
PTH: Parathyroid hormone
PTMC: Papillary microcarcinoma
RAI: Radioiodine
RFS: Recurrence-free survival
RR: Relative risk
SEER: Surveillance, Epidemiology and End Results
Tg: Thyroglobulin
TL: Thyroid lobectomy
TNM: Tumor Nodes Metastasis
TSH: Thyroid stimulation hormone
TT: Total thyroidectomy
UICC: Union for International Cancer Control

## References

[1] Pellegriti G, Frasca F, Regalbuto C, Squatrito S, Vigneri R (2013). Worldwide increasing incidence of thyroid cancer: update on epidemiology and risk factors. J Cancer Epidemiol.

[2] Davies L, Welch HG (2014). Current thyroid cancer trends in the United States. JAMA Otolaryngol Head Neck Surg.

[3] Krajewska J, Kukulska A, Oczko-Wojciechowska M (2020). Early Diagnosis of Low-Risk Papillary Thyroid Cancer Results Rather in Overtreatment Than a Better Survival. Front Endocrinol (Lausanne).

[4] Haugen BR, Alexander EK, Bible KC (2016). 2015 American Thyroid Association Management Guidelines for Adult Patients with Thyroid Nodules and Differentiated Thyroid Cancer: The American Thyroid Association Guidelines Task Force on Thyroid Nodules and Differentiated Thyroid Cancer. Thyroid.

[5] Twining CL, Lupo MA, Tuttle RM (2018). Implementing Key Changes in the American Thyroid Association 2015 Thyroid Nodules/Differentiated Thyroid Cancer Guidelines Across Practice Types. Endocr Pract.

[6] Cooper DS, Doherty GM, Haugen BR (2009). Revised American Thyroid Association management guidelines for patients with thyroid nodules and differentiated thyroid cancer. Thyroid.

[7] Kim BW, Yousman W, Wong WX, Cheng C, McAninch EA (2016). Less is More: Comparing the 2015 and 2009 American Thyroid Association Guidelines for Thyroid Nodules and Cancer. Thyroid.

[8] Luster M, Aktolun C, Amendoeira I (2019). European Perspective on 2015 American Thyroid Association Management Guidelines for Adult Patients with Thyroid Nodules and Differentiated Thyroid Cancer: Proceedings of an Interactive International Symposium. Thyroid.

[9] Filetti S, Durante C, Hartl D (2019). Thyroid cancer: ESMO Clinical Practice Guidelines for diagnosis, treatment and follow-up†. Ann Oncol Off J Eur Soc Med Oncol.

[10] Haddad R, Bischoff L, Bernet V, et al. NCCN Clinical Practice Guidelines in Oncology (NCCN Guidelines) Thyroid Carcinoma. Published 2021. https://www.nccn.org/professionals/physician_gls/pdf/thyroid.pdf. Accessed 4 Apr 2021.

[11] Samaan NA, Schultz PN, Hickey RC, et al. The results of various modalities of treatment of well differentiated thyroid carcinomas: a retrospective review of 1599 patients. J Clin Endocrinol Metab. 1992;75(3):714–20. http://www.ncbi.nlm.nih.gov/pubmed/1517360. Accessed 9 Sept 2013. 10.1210/jcem.75.3.1517360 1517360

[12] DeGroot LJ, Kaplan EL, McCormick M, Straus FH (1990). Natural history, treatment, and course of papillary thyroid carcinoma. J Clin Endocrinol Metab.

[13] Mazzaferri EL, Young RL (1981). Papillary thyroid carcinoma: a 10 year follow-up report of the impact of therapy in 576 patients. Am J Med.

[14] Hay ID, Thompson GB, Grant CS (2002). Papillary thyroid carcinoma managed at the Mayo Clinic during six decades (1940–1999): temporal trends in initial therapy and long-term outcome in 2444 consecutively treated patients. World J Surg.

[15] Shaha AR, Shah JP, Loree TR (1997). Differentiated thyroid cancer presenting initially with distant metastasis. Am J Surg.

[16] Sanders LE, Cady B (1998). Differentiated thyroid cancer: reexamination of risk groups and outcome of treatment. Arch Surg.

[17] Mendelsohn AH, Elashoff DA, Abemayor E, St John MA (2010). Surgery for papillary thyroid carcinoma: is lobectomy enough?. Arch Otolaryngol Head Neck Surg.

[18] Matsuzu K, Sugino K, Masudo K (2014). Thyroid lobectomy for papillary thyroid cancer: long-term follow-up study of 1,088 cases. World J Surg.

[19] Nixon IJ, Ganly I, Patel SG (2012). Thyroid lobectomy for treatment of well differentiated intrathyroid malignancy. Surgery.

[20] Haigh PI, Urbach DR, Rotstein LE (2005). Extent of thyroidectomy is not a major determinant of survival in low- or high-risk papillary thyroid cancer. Ann Surg Oncol.

[21] Adam MA, Pura J, Gu L, et al. Extent of surgery for papillary thyroid cancer is not associated with survival: an analysis of 61,775 patients. “PG - 601–5; discussion 605–7.” Ann Surg. 2014;260(4). 10.1097/SLA.0000000000000925 10.1097/SLA.0000000000000925 PMC4532384 25203876

[22] Barney BM, Hitchcock YJ, Sharma P, Shrieve DC, Tward JD (2011). Overall and cause-specific survival for patients undergoing lobectomy, near-total, or total thyroidectomy for differentiated thyroid cancer. Head Neck.

[23] Bilimoria KY, Bentrem DJ, Ko CY, et al. Extent of surgery affects survival for papillary thyroid cancer. “PG - 375–81; discussion 381–4.” Ann Surg. 2007;246(3). 10.1097/SLA.0b013e31814697d9 10.1097/SLA.0b013e31814697d9 PMC1959355 17717441

[24] Shah JP (2008). Re: Extent of surgery affects papillary thyroid cancer. Ann Surg.

[25] Kuba S, Yamanouchi K, Hayashida N (2017). Total thyroidectomy versus thyroid lobectomy for papillary thyroid cancer: Comparative analysis after propensity score matching: A multicenter study. Int J Surg.

[26] Song E, Han M, Oh HS (2019). Lobectomy Is Feasible for 1–4 cm Papillary Thyroid Carcinomas: A 10-Year Propensity Score Matched-Pair Analysis on Recurrence. Thyroid.

[27] Choi JB, Lee SG, Kim MJ (2019). Oncologic outcomes in patients with 1-cm to 4-cm differentiated thyroid carcinoma according to extent of thyroidectomy. Head Neck.

[28] Bojoga A, Koot A, Bonenkamp J, et al. The Impact of the Extent of Surgery on the Long-Term Outcomes of Patients with Low-Risk Differentiated Non-Medullary Thyroid Cancer: A Systematic Meta-Analysis. J Clin Med. 2020;9(7). 10.3390/jcm9072316 10.3390/jcm9072316 PMC7408649 32708218

[29] Bosset M, Bonjour M, Castellnou S (2021). Long-Term Outcome of Lobectomy for Thyroid Cancer. Eur Thyroid J.

[30] Matsuura D, Yuan A, Harries V, et al. Surgical Management of Low-/Intermediate-Risk Node Negative Thyroid Cancer: A Single-Institution Study Using Propensity Matching Analysis to Compare Thyroid Lobectomy and Total Thyroidectomy. Thyroid. Published online December 3, 2021. 10.1089/THY.2021.0356 10.1089/thy.2021.0356 PMC8792497 34861772

[31] Rajjoub SR, Yan H, Calcatera NA (2018). Thyroid lobectomy is not sufficient for T2 papillary thyroid cancers. Surgery.

[32] Zhang C, Li Y, Li J, Chen X. Total thyroidectomy versus lobectomy for papillary thyroid cancer: A systematic review and meta-analysis. Medicine (Baltimore). 2020;99(6). 10.1097/MD.0000000000019073 10.1097/MD.0000000000019073 PMC7015547 32028431

[33] Hirshoren N, Kaganov K, Weinberger JM (2018). Thyroidectomy Practice After Implementation of the 2015 American Thyroid Association Guidelines on Surgical Options for Patients With Well-Differentiated Thyroid Carcinoma. JAMA Otolaryngol Head Neck Surg.

[34] Adhami M, Bhatt CR, Grodski S, Serpell J, Lee JC (2021). Less extensive surgery for low-risk papillary thyroid cancers post 2015 American Thyroid Association guidelines in an Australian tertiary centre. Eur J Surg Oncol.

[35] Wrenn SM, Wang TS, Toumi A, Kiernan CM, Solórzano CC, Stephen AE (2021). Practice patterns for surgical management of low-risk papillary thyroid cancer from 2014 to 2019: A CESQIP analysis. Am J Surg.

[36] James BC, Timsina L, Graham R, Angelos P, Haggstrom DA (2019). Changes in total thyroidectomy versus thyroid lobectomy for papillary thyroid cancer during the past 15 years. Surgery.

[37] Bove A, Panaccio P, Palone G, Esposito L, Marino L, Bongarzoni G. Impact of the new guidelines of the American Thyroid Association on the treatment of the differentiated thyroid tumor in an Italian center with medium-high volume thyroid surgery. BMC Surg. 2019;18(Suppl 1). 10.1186/S12893-018-0462-8 10.1186/s12893-018-0462-8 PMC7402582 31074397

[38] Calò PG, Erdas E, Medas F (2015). Differentiated thyroid cancer: feasibility of loboisthmectomy in an endemic region. G Chir.

[39] Kiernan CM, Whiteside MA, Solorzano CC (2017). Cancer Registries: Can We Improve the Quality of Thyroid Cancer Data?. Ann Surg Oncol.

[40] Campennì A, Barbaro D, Guzzo M, Capoccetti F, Giovanella L (2020). Personalized management of differentiated thyroid cancer in real life - practical guidance from a multidisciplinary panel of experts. Endocrine.

[41] Sawant R, Hulse K, Sohrabi S (2019). The impact of completion thyroidectomy. Eur J Surg Oncol.

[42] DiMarco AN, Wong MS, Jayasekara J (2019). Risk of needing completion thyroidectomy for low-risk papillary thyroid cancers treated by lobectomy. BJS open.

[43] Shaha AR, Michael TR (2019). Completion thyroidectomy-indications and complications. Eur J Surg Oncol.

[44] Al Afif A, Williams BA, Rigby MH (2015). Multifocal Papillary Thyroid Cancer Increases the Risk of Central Lymph Node Metastasis. Thyroid.

[45] Leung AM, Dave S, Lee SL, Campion FX, Garber JR, Pearce EN (2011). Factors determining the persistence or recurrence of well-differentiated thyroid cancer treated by thyroidectomy and/or radioiodine in the Boston, Massachusetts area: A retrospective chart review. Thyroid Res.

[46] Woo J, Kim H, Kwon H (2021). Impact of Multifocality on the Recurrence of Papillary Thyroid Carcinoma. J Clin Med.

[47] Del Rio P, Loderer T, Giuffrida M (2021). Multifocality in patients treated for papillary Thyroid Carcinoma: a preliminary analysis of related risk factors. Acta Biomed.

[48] Kim SK, Park I, Woo JW (2017). Predicting Factors for Bilaterality in Papillary Thyroid Carcinoma with Tumor Size. Thyroid.

[49] Zhang F, Zheng B, Yu X, Wang X, Wang S, Teng W (2021). Risk Factors for Contralateral Occult Carcinoma in Patients With Unilateral Papillary Thyroid Carcinoma: A Retrospective Study and Meta-Analysis. Front Endocrinol (Lausanne).

[50] Grigsby PW, Reddy RM, Moley JF, Hall BL (2006). Contralateral papillary thyroid cancer at completion thyroidectomy has no impact on recurrence or survival after radioiodine treatment. Surgery.

[51] Harries V, Wang LY, McGill M (2020). Should multifocality be an indication for completion thyroidectomy in papillary thyroid carcinoma?. Surgery.

[52] Bychkov A. Thyroid and parathyroid. Thyroid general AJCC / TNM staging. Pathology Outlines. https://www.pathologyoutlines.com/topic/thyroidstaging.html. Accessed 15 Sept 2021.

[53] Diker-Cohen T, Hirsch D, Shimon I, et al. Impact of Minimal Extra-Thyroid Extension in Differentiated Thyroid Cancer: Systematic Review and Meta-analysis. J Clin Endocrinol Metab. Published online March 1, 2018. 10.1210/jc.2018-00081 10.1210/jc.2018-00081 29506045

[54] Park YM, Lee DY, Oh KH (2017). Clinical implications of pathologic factors after thyroid lobectomy in patients with papillary thyroid carcinoma. Oral Oncol.

[55] Shin JH, Ha TK, Park HK (2013). Implication of minimal extrathyroidal extension as a prognostic factor in papillary thyroid carcinoma. Int J Surg.

[56] Weber M, Binse I, Oebbecke K (2021). Analysis of risk factors and prognosis in differentiated thyroid cancer with focus on minimal extrathyroidal extension. BMC Endocr Disord.

[57] Tam S, Amit M, Boonsripitayanon M (2018). Effect of Tumor Size and Minimal Extrathyroidal Extension in Patients with Differentiated Thyroid Cancer. Thyroid.

[58] Amit M, Boonsripitayanon M, Goepfert RP (2018). Extrathyroidal Extension: Does Strap Muscle Invasion Alone Influence Recurrence and Survival in Patients with Differentiated Thyroid Cancer?. Ann Surg Oncol.

[59] Cox C, Bosley M, Southerland LB (2018). Lobectomy for treatment of differentiated thyroid cancer: can patients avoid postoperative thyroid hormone supplementation and be compliant with the American Thyroid Association guidelines?. Surgery.

[60] Wilson M, Patel A, Goldner W, Baker J, Sayed Z, Fingeret AL (2020). Postoperative thyroid hormone supplementation rates following thyroid lobectomy. Am J Surg.

[61] Xiao L, Wu J, Jiang L, Xu Y, Liu B. Is thyroid hormone supplementation avoidable for patients with low-risk papillary thyroid cancer after thyroid lobectomy? A two-center observational study. Clin Endocrinol (Oxf). Published online 2021. 10.1111/CEN.14580 10.1111/cen.14580 34398464

[62] Lee SJ, Song CM, Ji YB (2021). Risk factors for hypothyroidism and thyroid hormone replacement after hemithyroidectomy in papillary thyroid carcinoma. Langenbeck’s Arch Surg.

[63] Beisa V, Kazanavicius D, Skrebunas A, Simutis G, Ivaska J, Strupas K. Prospective analysis of risk for hypothyroidism after hemithyroidectomy. Int J Endocrinol. 2015;2015. 10.1155/2015/313971 10.1155/2015/313971 PMC4396907 25918526

[64] Zatelli MC, Lamartina L, Meringolo D (2018). Thyroid nodule recurrence following lobo-isthmectomy: incidence, patient’s characteristics, and risk factors. J Endocrinol Invest.

[65] Dou Y, Chen Y, Hu D, Su X. The Recovery of Thyroid Function in Low-Risk Papillary Thyroid Cancer After Lobectomy: A 3-Year Follow-Up Study. Front Endocrinol (Lausanne). 2021;11. 10.3389/FENDO.2020.619841 10.3389/fendo.2020.619841 PMC7899978 33633689

[66] Kim SY, Kim HJ, Kim SM, et al. Thyroid Hormone Supplementation Therapy for Differentiated Thyroid Cancer After Lobectomy: 5 Years of Follow-Up. Front Endocrinol (Lausanne). 2020;11. 10.3389/FENDO.2020.00520 10.3389/fendo.2020.00520 PMC7412630 32849303

[67] Loyo M, Tufano RP, Gourin CG (2013). National trends in thyroid surgery and the effect of volume on short-term outcomes. Laryngoscope.

[68] Stavrakis AI, Ituarte PHG, Ko CY, Yeh MW (2007). Surgeon volume as a predictor of outcomes in inpatient and outpatient endocrine surgery. Surgery.

[69] Kandil E, Noureldine SI, Abbas A, Tufano RP (2013). The impact of surgical volume on patient outcomes following thyroid surgery. Surgery.

[70] Gourin CG, Tufano RP, Forastiere AA, Koch WM, Pawlik TM, Bristow RE (2010). Volume-based trends in thyroid surgery. Arch Otolaryngol Head Neck Surg.

[71] Hauch A, Al-Qurayshi Z, Randolph G, Kandil E (2014). Total thyroidectomy is associated with increased risk of complications for low- and high-volume surgeons. Ann Surg Oncol.

[72] Nouraei SAR, Allen J, Kaddour H (2017). Vocal palsy increases the risk of lower respiratory tract infection in low-risk, low-morbidity patients undergoing thyroidectomy for benign disease: A big data analysis. Clin Otolaryngol.

[73] Gunn A, Oyekunle T, Stang M, Kazaure H, Scheri R (2020). Recurrent Laryngeal Nerve Injury After Thyroid Surgery: An Analysis of 11,370 Patients. J Surg Res.

[74] Godlewska P, Benke M, Stachlewska-Nasfeter E, Gałczyński J, Puła B, Dedecjus M (2020). Risk factors of permanent hypoparathyroidism after total thyroidectomy and central neck dissection for papillary thyroid cancer: a prospective study. Endokrynol Pol.

[75] Bergenfelz A, Nordenström E, Almquist M (2020). Morbidity in patients with permanent hypoparathyroidism after total thyroidectomy. Surgery.

[76] Almquist M, Ivarsson K, Nordenström E, Bergenfelz A (2018). Mortality in patients with permanent hypoparathyroidism after total thyroidectomy. Br J Surg.

[77] Annebäck M, Hedberg J, Almquist M, Stålberg P, Norlén O (2020). Risk of Permanent Hypoparathyroidism After Total Thyroidectomy for Benign Disease: A Nationwide Population-based Cohort Study From Sweden. Ann Surg.

[78] Nordenström E, Bergenfelz A, Almquist M (2018). Permanent Hypoparathyroidism After Total Thyroidectomy in Children: Results from a National Registry. World J Surg.

[79] Neagoe RM, Cvasciuc IT, Muresan M, Sala DT. Incidental parathyroidectomy during thyroid surgery - risk, prevention and controversies; an evidence-based review. Acta Endocrinol (Bucharest, Rom 2005). 2017;13(4):467–75. 10.4183/AEB.2017.467 10.4183/aeb.2017.467 PMC6516554 31149218

[80] Almquist M, Hallgrimsson P, Nordenström E, Bergenfelz A (2014). Prediction of permanent hypoparathyroidism after total thyroidectomy. World J Surg.

[81] Lorek AJ, Steinhof-Radwańska K, Zarębski W (2021). The prevalence of hypoparathyroidism after thyroid surgery depending on the diagnosis, the extent of the procedure, and the presence of parathyroid glands in the postoperative examination. Endokrynol Pol.

[82] Bai B, Chen Z, Chen W (2018). Risk factors and outcomes of incidental parathyroidectomy in thyroidectomy: A systematic review and meta-analysis. PLoS One.

[83] Frey S, Figueres L, Pattou F (2021). Impact of Permanent Post-thyroidectomy Hypoparathyroidism on Self-evaluation of Quality of Life and Voice: Results From the National QoL-Hypopara Study. Ann Surg.

[84] Büttner M, Hinz A, Singer S, Musholt T (2020). Quality of Life of Patients More Than 1 Year After Surgery for Thyroid Cancer. Hormones (Athens).

[85] Momesso DP, Vaisman F, Yang SP (2016). Dynamic Risk Stratification in Patients with Differentiated Thyroid Cancer Treated Without Radioactive Iodine. J Clin Endocrinol Metab.

[86] Park S, Jeon MJ, Oh HS (2018). Changes in Serum Thyroglobulin Levels After Lobectomy in Patients with Low-Risk Papillary Thyroid Cancer. Thyroid.

[87] Ritter A, Mizrachi A, Bachar G (2020). Detecting Recurrence Following Lobectomy for Thyroid Cancer: Role of Thyroglobulin and Thyroglobulin Antibodies. J Clin Endocrinol Metab.

[88] Marciniak C, Lenne X, Clément G (2021). Partial Versus Total Thyroidectomy: What Influences Most Surgeons’ Decision? Analysis of a Nationwide Cohort of 375,810 Patients Over 10 Years. Ann Surg.

[89] Sosa JA, Bowman HM, Tielsch JM, Powe NR, Gordon TA, Udelsman R (1998). The importance of surgeon experience for clinical and economic outcomes from thyroidectomy. Ann Surg.

[90] McDow AD, Saucke MC, Marka NA, Long KL, Pitt SC (2021). Thyroid Lobectomy for Low-Risk Papillary Thyroid Cancer: A National Survey of Low- and High-Volume Surgeons. Ann Surg Oncol.

[91] Lang BHH, Wong CKH (2016). Lobectomy is a more Cost-Effective Option than Total Thyroidectomy for 1 to 4 cm Papillary Thyroid Carcinoma that do not Possess Clinically Recognizable High-Risk Features. Ann Surg Oncol.

[92] Grani G, Cera G, Conzo G (2021). Preoperative Ultrasonography in the Evaluation of Suspected Familial Non-Medullary Thyroid Cancer: Are We Able to Predict Multifocality and Extrathyroidal Extension?. J Clin Med.

[93] Dhir M, McCoy KL, Ohori NP (2018). Correct extent of thyroidectomy is poorly predicted preoperatively by the guidelines of the American Thyroid Association for low and intermediate risk thyroid cancers. Surgery.

[94] Kluijfhout WP, Pasternak JD, Lim J (2016). Frequency of High-Risk Characteristics Requiring Total Thyroidectomy for 1–4 cm Well-Differentiated Thyroid Cancer. Thyroid.

[95] Bakkar S, Al-Omar K, Donatini G (2021). Postoperatively determined high-risk histopathologic features in papillary thyroid carcinoma initially eligible for thyroid lobectomy: a game changer. Endocrine.

[96] Lang BHH, Shek TWH, Wan KY (2017). The significance of unrecognized histological high-risk features on response to therapy in papillary thyroid carcinoma measuring 1–4 cm: implications for completion thyroidectomy following lobectomy. Clin Endocrinol (Oxf).

[97] Craig SJ, Bysice AM, Nakoneshny SC, Pasieka JL, Chandarana SP (2020). The Identification of Intraoperative Risk Factors Can Reduce, but Not Exclude, the Need for Completion Thyroidectomy in Low-Risk Papillary Thyroid Cancer Patients. Thyroid.

[98] van Gerwen M, Cooke PV, Alpert N, Mols F, Genden E, Schwartz RM (2022). Patient-reported outcomes following total thyroidectomy and lobectomy in thyroid cancer survivors: an analysis of the PROFILES Registry data. Support Care Cancer.

[99] Nickel B, Tan T, Cvejic E (2019). Health-Related Quality of Life After Diagnosis and Treatment of Differentiated Thyroid Cancer and Association With Type of Surgical Treatment. JAMA Otolaryngol Head Neck Surg.

[100] Ahmadi S, Gonzalez JM, Talbott M (2020). Patient Preferences Around Extent of Surgery in Low-Risk Thyroid Cancer: A Discrete Choice Experiment. Thyroid.

[101] Lubitz CC, Kiernan CM, Toumi A (2021). Patient Perspectives on the Extent of Surgery and Radioactive Iodine Treatment for Low-Risk Differentiated Thyroid Cancer. Endocr Pract.

[102] McDow AD, Roman BR, Saucke MC (2021). Factors associated with physicians’ recommendations for managing low-risk papillary thyroid cancer. Am J Surg.

